# The future of plant biotechnology in a globalized and environmentally endangered world

**DOI:** 10.1590/1678-4685-GMB-2019-0040

**Published:** 2019-12-20

**Authors:** Marc Van Montagu

**Affiliations:** 1 VIB-International Plant Biotechnology Outreach, Ghent University, Ghent, Belgium

**Keywords:** Plant biotech, GMO, sustainable agriculture, science and society

## Abstract

This paper draws on the importance of science-based agriculture in order to throw light on the way scientific achievements are at the basis of modern civilization. An overview of literature on plant biotechnology innovations and the need to steer agriculture towards sustainability introduces a series of perspectives on how plant biotech can contribute to the major challenge of feeding our super population with enough nutritious food without further compromise of the environment. The paper argues that science alone will not solve problems. Three major forces - science, the economy and society - shape our modern world. There is a need for a new social contract to harmonize these forces. The deployment of the technologies must be done on the basis of ethical and moral values.

## Introduction

Agriculture is probably the biggest early success of human ingenuity. It initiated the development of environmental changes without which we would not exist as a present-day society, for the best and worst. Humans have continuously improved agriculture since the dawn of civilization, wheat being the first domestication recorded by historians some 9,000 years ago. Human civilizations have spread agriculture far and wide, and, as some call it, “manipulation of species” by early agriculturalists is the foundation of modern agriculture. The application in our lifetime of the genetic knowledge for crop improvement has led to unprecedented growth in agricultural productivity. It is largely recognized that without these advances in plant breeding, global food shortages would be a much more critical issue today. But it is not enough.

Although it is true that per capita agricultural production of current intensive agriculture has outpaced population growth, the rich of the world reap most of the benefits. Moreover, externalities such as climate change are expected to offset the positive effect of economic growth on food security. Climate change will not only compromise food security but also food safety, by increasing food-borne pathogens or inducing chemical changes that can increase the prevalence of toxic compounds in food (FAO, 2018). At the same time, as we learn about the value of soil microorganisms to agriculture, we understand the collateral damage of crop protection chemicals.

The issue of food security and safety is a global issue that affects the whole food system. Questions arise on how to change food habits and ways to reduce waste in the food chain, from harvest to the moment of consumption. Although it is important to tackle these issues, it does not change the fact that we will need to change our dominant agricultural model in order to feed a growing global population in a way compatible with the sustainable use of global resources. Innovative agriculture and food systems must be tailored to a diverse global population whilst preserving the variety of its cultures i.e., fitting the characteristics and needs of various individuals, cultures, and social groups.

Our societies have managed to keep war, pandemics, and famine at manageable levels thanks to the technological, economic and political developments we brought about in the past decades. Today, biotechnology developments are delivering an array of powerful tools to medicine and agriculture. Physics and chemistry, associated with information technology are providing us with a power unimaginable before.

Take a moment to observe how the world is changing: fueled by information and communication technology we have generated a global flow of networks of activity and interaction that has integrated the global economy, media, legal practices, and scientific research. Globalization is promoting a steady integration of different societies and customs. At the same time, the global society is about to change radically. Routine skills used by the industrial revolution are no longer sufficient as we are approaching the post-digital transformation. Disruptive technologies such as AI (artificial intelligence), robotics, IoT (internet of things), and blockchain have the potential of transforming business models, companies and jobs. Although these technologies have great potential to change the world for the better, an uneasy dystopian climate of opinion is growing. Many among us worry: Is it really going to be for the best? Aren’t the negative outcomes outpacing the benefits? Are we ready for these changes? How well do we understand life science?

As well observed by Juma (2016), technology, economy and society coevolve as a whole. Technological transformation must be followed by adequate institutional adjustments to avoid rupture of the social tissue. It will require a new social contract to harmonize the interest of all parties. The deployment of technologies must be done according to society’s ethical and moral values. In humans, the process of decision making uses both rational arguments and emotions. Emotional thinking can help us to make judgments in an uncertain environment. Till very recently, non-verifiable arguments as well as emotional thinking were the only tools we had to recognize a problem and try to solve it. Starting from the Enlightenment, Western society slowly moved to scientific, fact-based arguments as the main support for decision making. Emotional thinking is nonetheless a powerful force for decision making as well. The problem has ethical roots. If emotions are manipulated by unethical pressure groups or problematic scientific dissidents, they can block a worthy innovative technology.

## Plant biotechnology is a mature technology

Modern biotechnology was a scientifically obvious outcome of the striking advances in molecular biology that followed the discovery of the bacterial DNA restriction-modification system (Luria and Human, 1952; Luria, 1953; Dussoix and Arber, 1962; Nathans and Smith, 1975). Microorganisms and plants were the first organisms to be manipulated to serve humankind. In the field of plant sciences, biotechnology was possible thanks to the discovery of *Agrobacterium tumefaciens’* Ti plasmid and its role in the natural bacteria-plant transgenesis (for historical review see Van Montagu, 2011; Chilton, 2018; Heimann, 2018).

Plant biotechnology with focus on seed-varietal improvement, such as GM technology and molecular-assisted breeding, has generated products that help agriculture to achieve enhanced yields in a more sustainable manner. Since the proof-of-concept in tobacco plants, the number of plant species with GM varieties approved worldwide increased sharply. As of January 2019, a total of 44 countries granted regulatory approvals to 40 GM crops and 509 GM events, covering 41 GM commercial traits for use in food, feed and/or for cultivation (ISAAA’s GM Approval Database, 2019). Approved GM varieties include food/feed crops (maize, rice, soybean, colza, wheat, bean, chicory, eggplant, tomato, sweet pepper, flax, potato, squash, apple, melon, papaya, plum), cash crops (cotton, sugar beet, sugar cane, creeping bent grass, safflower, tobacco), ornamental plants (petunia, carnation, rose), and forestry trees (eucalyptus, poplar). The GM traits are numerous and diverse. A non-exhaustive list spans from input traits (herbicide tolerance, insect resistance, drought stress tolerance, bacterial and virus disease resistance), to output traits to improve yield (enhanced photosynthesis, increased ear biomass), product quality (anti-allergy, delayed fruit softening, delayed ripening/senescence, enhanced provitamin A, lowered reducing sugars, mannose metabolism, modified starch/carbohydrate, modified amino acid, modified oil/fatty acid, nicotine reduction, non-browning phenotype, altered lignin production, volumetric wood increase, modified flower colour), and pollination control (male sterility, fertility restoration).

GM-technology is largely considered the technology that most affected agriculture in recent times. Indeed, by 2017, the global adoption of GM crops reached 189.8 million hectares (ISAAA, 2017). The striking amount of approved GM varieties and hybrids shows that GM technology does not narrow the genetic diversity of the crop plant.

For over 20 years humans and animals have been eating GM food of different types without ill effects.Whereas nobody can ever say that anything, including any food, is safe, the evidence of GM consumption and use in massive quantities validate the premise that GM crops are at least as safe as any non-GM crop. A number of meta-analyses of peer-reviewed scientific publications across two decades of commercialization confirm that the GM crops pose no risk to human and livestock health (e.g., Swiatkiewicz *et al.*, 2014; de Vos and Swanenburg, 2018; Pellegrino *et al.*, 2018).

The scientific consensus is that there is no evidence of hazards in the movement of genes between unrelated organisms, or in the use of recombinant DNA techniques. Respected scientific organizations such as World Health Organization, the American Medical Association, the U.S. National Academy of Sciences, the British Royal Society have come to the same conclusion after a careful scrutinization of the evidence: “*consuming foods containing ingredients derived from GM crops is no riskier than consuming the same foods containing ingredients from crop plants modified by conventional plant improvement techniques*”. The analysis of Pellegrino *et al.* (2018) on two decades of GM maize consumption not only confirmed that GM maize pose no risk to human or livestock health, but also showed that GM insect resistant varieties could have a substantive positive impact on human and livestock health. This is because insects weaken the plant’s immune system. Transgenic maize with decreased insect damage is less susceptible to fungal infection. Hence, biotech maize contains substantially fewer mycotoxins. Mycotoxins are both toxic and carcinogenic to humans and animals.

Scientists have also scrutinized the environmental safety of GM crops. The EU has invested more than 300m EUR in more than 130 research projects involving 500 independent groups, covering a period of more than 25 years of research to arrive at the conclusion that “*biotechnology, and in particular GMOs, are not per se more risky than e.g. conventional plant breeding technologies*.” (European Commission, 2010). In fact, GM crops with input traits for insect resistance and herbicide tolerance have contributed to reduce agriculture’s environmental footprint by facilitating environmentally friendly farming practices (Brookes and Barfoot, 2015). Klumper and Qaim (2014) conducted a meta-analysis based on primary data from farm surveys or field trials in different regions worldwide. This comprehensive study demonstrates that GM insect resistant (IR) traits have reduced pesticide usage by 36.9% on average.

GM herbicide tolerance (HT) traits also bring substantial contribution to sustainable agriculture. GM HT allows the application of more environmentally friendly herbicide (e.g., glyphosate) in a more rational way and enables the adoption of conservation tillage. Sowing seeds directedly into the fields without previous ploughing preserves beneficial soil insects and earth worms, retains soil moisture - which is good for water conservation - and keeps carbon in the soil. Abdalla *et al.* (2016) carried out a meta-analysis of peer-reviewed publications comparing CO_2_ emissions over entire seasons or years from tilled and untilled soils, across different climates, crop types, and soil conditions. The authors concluded that, on average, tilled soils emitted 21% more CO_2_ than untilled soils. Moreover, less pesticide and no/less ploughing have also reduced the use of powered agricultural machines. Less tractor traffic causes indirect benefits to soil quality, conserves fossil fuel, and decreases CO_2_ emissions to the atmosphere.

Habitat destruction is the biggest single threat to biodiversity. The higher productivity of the currently commercialized GM crops alleviates the pressure to convert additional land for agriculture. For example, if the world were no longer to use GM crops, an additional 22.4 Mha would be required to maintain the global production at 2016 levels (Brookes and Barfoot, 2018). For GM HT crops alone, the land use impact would be more 762 Mha of cropping area, of which 53% would be new land brought into agriculture, including 167 Mha of deforestation. Besides a major impact on wildlife habitats, the increase in cropping area would generate 234 billion kg more of CO_2_ emissions (Brookes *et al.*, 2017)

Farmers’ acceptance is impressive; when given the opportunity, they quickly adopted GM crops. Since the introduction of the technology in the mid 1990s till 2016, 18 million farmers planted biotech crops (ISAAA, 2017). Biotech crops have a historical track record of economic benefits, logistical advantages, and risk reductions. Twenty-one years of GM crop agriculture produced a net economic benefit at farm level of $ 186.1 billion, of which 52% were reaped by farmers in developing countries. These gains are mostly yield and productivity gains (65%); the remaining 35% are from cost savings (Brookes and Barfoot, 2018).

Although impressive, the outcome of GM crops is far below what it could be. Only a dozen genetically modified crops are available today, of which four (soybean, maize, cotton, and canola), carrying only two traits (herbicide tolerance and insect resistance) out of 41 that have been approved, occupy 99.2% of the global GM planted area. The vast majority of approved GM varieties are kept on the laboratory shelf.

Nonetheless, sound R&D projects continue to be carried out, thanks to the advances in plant molecular biology and genome sequencing at low costs. Many of these projects are being developed in and for low income countries, particularly in Africa, often through collaborative consortiums between public institutions, philanthropic organizations, and agrobusiness corporations. A wide variety of plants is being made to be resilient to biotic and abiotic stresses, to have increased water or nitrogen use efficiency (NUE), and nutritional improvements (Ricroch and Hénard-Damave, 2016). Other relevant innovations for non-food purposes, such as biopharmaceuticals, biofuel, starch, paper, and textile industries are progressing in developed countries (De Buck *et al.*, 2016).

Traditionally, breeding strategies have been focused on increasing crop productivity through yield increase and disease and pest resistance. Breeders have often neglected the nutritional value of food crops. The outcome of this tactic was the rise of micronutrient malnutrition or “hidden hunger”, particularly in food-insecure regions, where diets are dominated by staple food crops. Food biofortification is of particular interest for low income countries. Microminerals and vitamins regulate important metabolic processes that play crucial roles in human physical and mental development. Childhood stunting is associated with micronutrient malnutrition in children, starting from fetal development to four years of age (FAO, IFAD, UNICEF, WFP and WHO, 2018).

Plant biotechnology is the only alternative for engineering metabolic pathways to improve micronutrients in a crop where they do not occur naturally. Furthermore, a given GM biofortification can be replicated to multiple target crops (Garg *et al.*, 2018). There have been significant advances in the development of GM biofortified plants. Numerous crops have been engineered to enhance the contend in vitamins, minerals, essential amino acids, and essential fatty acids; Golden Rice being the best known-example. The same or similar strategies used in Golden Rice have been used with success to engineer pro-VitA in different crops such as banana, cassava, potato, sorghum, soybean, and sweet potato. Reports are available for biofortified cereals, legumes, vegetables, oilseeds, fruits, and fodder crops. Successful examples include high lysine maize, high unsaturated fatty acid soybean, and iron and zinc rich cassava, folate rich rice (Garg *et al.*, 2018). None have entered the commercialization phase in low income countries, where they are mostly needed.

Biotechnology tools are constantly evolving. New powerful technologies for gene editing (Doudna and Charpentier, 2014) are now made available for plant amelioration and are expected to revolutionize the breeding programs in the near future. These so-called new breeding techniques are likely to be applied in the amelioration of a wider variety of plants, boosting the germplasm resource for agriculture worldwide. Genome editing will greatly facilitate the engineering of complex traits, such as stacked disease tolerance and insect resistance mechanisms, resilience to abiotic stress, as well as nutritional and organoleptic properties (Halewood, 2018). Besides disrupting gene function, or editing existing sequences to reproduce ancient alleles, the technology allows the introduction of novel alleles or any other novel genetic material. Some recent remarkable examples of the potential of precision genome engineering for crop improvement are the *de novo* domestication of wild tomato, a showcase on how to exploit the genetic diversity of wild plants (Zsögön *et al.*, 2018), and the engineering of apomix in rice to produce hybrids (Khanday *et al.*, 2018).

A very interesting genome editing target are epigenetic markers, such as histone modification and DNA methylation. Epigenetics has emerged as a new way of regulating cellular functions in plants, as epigenetic changes are essential to adaptation to the environment. Modification of plant epigenomic patterns can be very useful to develop crops tolerant to environmental stresses such as drought and salinity. Although such research is still in its infancy, the first bricks have been laid, paving the way for the next generation of breeding. Lowder *et al.* (2015) successfully targeted a methylated promoter to activate an imprinted gene in *A. thaliana*, showing that it is possible to modify epigenetic markers to modulate gene expression.

Genome editing is quicker and cheaper than other techniques for crop improvement, such as induced mutagenesis and even transgenesis. Therefore, it can be a major game changer for agriculture in environmentally fragile regions, where many crops of local interest are niche-specific and well adapted to local environment and farming system. These crops have not yet received much attention by the scientific community for amelioration, because they represent only a small fraction of the international commodity trade (Varshney *et al.*, 2012).

The above overview allows us to assert that plant technology is a mature technology, safe, and with an extraordinary record of benefits, both economic and humanitarian. Genetic engineering and genome editing are ready to be deployed to improve crop breeding and build a more sustainable global agriculture.

## Why should we use plant biotechnology?

### Future farming system

*Homo sapiens* has altered Earth environments since its emergence as a species – probably as other species did, since life and environment are one. Environmental perturbations caused by humankind have evolved continuously since the beginning of civilization some 12,000 years ago. But, in the past century or two, we have changed ecosystems with such intensity, on such a scale, and such speed that a new geological era, the Anthropocene, has been proposed. The impact of mankind in Earth ecosystems is destabilizing the warm period of the past 10-12 millennia (Holocene), which is the only state of the planet that we know for sure can support contemporary human societies (Crutzen, 2002). As the global population expands to 10billion people, the pace of change is faster than ever before. There is an urgent need of a paradigm shift to maintain Earth in a safely operating space for humanity and for the millions of species with which we share this home. The dramatic negative impact that the massive scale of deforestation has caused not only to the habitat for millions of species, but also in overshooting key Earth system parameters (Steffen *et al.*, 2015). Undeniably, deforestation and intensive agriculture with `traditional’ crops are major human risk factors pushing the Earth system beyond the bounds of safety.

Although our inventiveness is the driver of the global problems we face, it is also the source of innovative solutions. The United Nations 2030 Agenda for Sustainable Development represents an important framework for tackling challenges. The UN 2030 agenda acknowledges that sustainable development goals (SDGs) cannot progress without strong engagement by science. Indeed, the robust knowledge-creation of the recent decades shows that science is accelerating its pace to bring the solutions that society is asking for. The transformative steps which are needed to shift the world onto a sustainable and resilient path need not leave anyone behind. The engagement by science - including the economic and social sciences - in the SDGs must involve all countries, developed and developing alike (UN Sustainable Developmental Goals).

Agriculture plays a crucial role in the SDGs because it is a main human activity that permeates most of the SDGs, from hunger and malnutrition to poverty alleviation, education, gender equality, water use, energy use, sustainable consumption and production, climate change, and ecosystem management. Agriculture acts as an engine of overall economic growth and development in many economies, notably in Least Developed Countries, where the majority of the poor are rural people. Global agriculture has been successful in providing sufficient food for expanding populations and their changing consumption patterns over recent decades. Per capita agricultural productivity has outpaced population growth. The steady long-term decline in real commodity prices attests to the success of the current dominant agricultural model. Intensification, rather than the spread of agricultural land, has been the prime driver of global agriculture productivity since the mid twentieth century (Pretty and Bharucha, 2014). Agriculture intensification was the only way forward, with the scientific knowledge then available, to cope with the dramatical rise in population in recent centuries. Between 1900 and 2000, the increase in world population was three times greater than during the entire previous history of humanity — an increase from 1.5 to 6.1 billion in just 100 years (Our World in Data). But this agriculture intensification has been accomplished at great expense to the environment, causing water scarcity, soil degradation, ecosystem stress, biodiversity loss, high levels of greenhouse gas emissions, and a significant decrease in forest cover.

Livestock is the world’s largest user of land resources. Grazing land and cropland dedicated to the production of animal feed represents almost 80% of all agricultural land (FAO-a). Agriculture and food production currently account for about 30% of energy consumption and about one-third of greenhouse gases (FAO-b). Agriculture also accounts for 40% of the Earth’s land surface, 70% of the world’s fresh water, with predictions that irrigation demands will increase by up to 100% by 2050 (UN Sustainable Developmental Goals). During the 20th century, the area under irrigation and the number of agricultural machines grew about two-fold, fertilizer consumption by four-fold, and nitrogen fertilizers by seven-fold (Pretty and Bharucha, 2014). The world consumed 186.67 million tons of fertilizers in 2016 (FAO-c), and about 6.8 million tons of pesticides in 2017 (IndexBox). Global fertilizers and agricultural chemicals manufacturing industry reached a revenue of $ 377 billion in 2018 (IbisWorld). Meanwhile, programs to control exposures to pesticides are limited or non-existent in several developing countries and as many as 25 million agricultural workers worldwide experience unintentional pesticide poisonings each year (Alavanja, 2009).

Allow me here a few words about glyphosate, the world’s most widely used active ingredient in herbicides and possibly the most heavily debated plant protection product. Recently, both EU and US biosafety agencies, concluded that human health risk levels associated with glyphosate exposure from food, drinking water, and residential sources are below levels of concern. Notwithstanding, both in Europe and the United States, these decisions were met by expressed public concerns about the possible risks of chemical exposures and the role of large multinational companies (van Straalen and Legler, 2018). Glyphosate illustrates a fundamental societal issue. Concerns about the control of food system by big agrobusiness companies influence people’s acceptance of scientific facts that attest to the safety of the product, and blinds some to the benefits of glyphosate for the environment. Anyway, the intensive agriculture concept of “getting more for more” – i.e., to produce as much biomass as possible with vast monocultures dependent on irrigation systems, fertilizers, and pesticides – is not acceptable any more. Yet transformation of agriculture is likely to be the greatest challenge of the UN 2030 Agenda for Sustainable Development.

Debates over the future of agriculture are being framed in two different ways: organic agriculture and sustainable intensification of agriculture. Organic farming relies on the use of natural inputs and ecological processes to make farms more sustainable, and deliberately excludes the use of external chemical inputs, such as fertilizers and synthetic pesticides, as well as GM crops. Organic agriculture practices are followed by 2.4 million farmers in about 87 countries that observe some sort of regulation or certification. It accounts to only 1.1% of the global agricultural land (Willer and Lernoud, 2017). Sustainable intensification (SI) takes the best idea of both conventional agriculture and organic farming. SI emphasizes the use of locally adapted management systems and gives preference to in-farm inputs (Timsina, 2018).

The potential for organic agriculture to feed the world is very debatable. Comparisons of yield data between organic and conventional agriculture report a lower yield for organic agriculture from 8% to 50% (reviewed by Timsina, 2018). Some studies suggest that adoption of organic agriculture under conditions of optimal performance might close the yield gap between organic and conventional crops. But these findings have been fiercely refuted by numerous scientists who claim faulty methodology. According to Connor (2018), “*one limitation alone, however, is sufficient to disqualify the notion of feeding the world organically, and that is the supply of nitrogen (N).*” The replacement of soil N removed by the product cannot be postponed without yield penalty. In organic fields, N must be supplied by *in situ* biological N fixation (BNF) of intercropped/rotated legumes and by *ex situ* BNF through use of organic material (manures, crops residues, green manures, bio fertilizers, etc.). Advocates of organic fertilizer often claim that there is enough organic material to supply the amounts as per the crop demand for high yield. The facts, though, are a bit different. First, organic materials are not universally available in large quantities. Second, when estimating organic productivity, organic farming supporters do not properly acknowledge the land that must be allocated – to both *in situ* and *ex situ* BNF - to supply nitrogen for the growth of non-legume crops. Alas, Lavoisier’s law also applies here. The data available clearly suggest that extra land and water would be needed if such sources of N were to be promoted (Timsina, 2018). By using more land to produce the same yield, current organic practices may ultimately accrue larger environmental costs.

The SI strategy is “to get more from less”. It seeks to produce the biomass needed by the growing human population on less land, with less adverse impact on the environment. SI does not specify particular technologies or practices. In particular, it focuses on increasing yields of farmland as a way to spare forest and other uncultivated land. A recent comprehensive study of the effect of land sparing that includes 17 organizations around the world concluded that more intensive agriculture that uses less land can be one way forward. The study found that inorganic nitrogen improved yields without increasing greenhouse gas. Intensification also produces fewer pollutants, causes less soil loss, and consumes less water (Balmford *et al.*, 2018). A global assessment for SI estimates that 163 million farms (29% of all worldwide) is practicing some form of SI on 453 Mha of agricultural land (9% of worldwide total) (Pretty *et al.*, 2018). Yet, SI acknowledges that there is no perfect solution due to the multi-objective nature of sustainability. The process is dynamic, and the technologies and practices will not fit everywhere and forever. For example, population growth is shifting to Africa that is projected to equal the Asian population in 2100 (United Nations, Department of Economic and Social Affairs, Population Division, 2017). Africa needs new sustainable intensification approaches to deal with the challenge of feeding an exponentially growing population. The solution will not come only from new management strategies. It is clear that the use of proper crop variety is the ultimate solution. Improved crop varieties suited to local conditions and weather extremes, as well as pest and disease resistant cultivars, increase yields and reduce pesticide use. As such, they are indispensable for SI ambition.Plant biotechnology has the tools to tailor crop varieties to the environment and help to meet the goal of producing more food without degrading ecosystems.

Under the umbrella of SI, management systems are developed to build on-farm soil fertility and *in situ* nutrient application. Sharing crop/pasture land with complementary species that do not compete for resources has been praised as a “win-win” management approach. Trees are ideal partners for crops, because they do not compete for the same source of nutrients. Tree roots go deeper into the soil and get nutrients and water from sources that are unavailable to crop species (Smith *et al.*, 2013). Well-designed agroforestry systems for sustainable intensification represent indeed an interesting sustainable strategy, particularly in those areas where the need for landscape restoration is associated with the need for increased food and biomass production. A rich scientific literature demonstrates the multiple benefits of this approach (reviewed by Timsina, 2018). Agroforestry does not require extra land because trees are planted around and among crops and pastures. Depending upon which woody species are used and how they are managed, agroforestry can build-up on-farm soil fertility, increasing resource-use efficiency, whilst reducing nutrient runoff. Research in Africa has demonstrated that the integration of fertilizer trees and shrubs into conventional agriculture can dramatically enhance soil fertility and food production (Garrity *et al.*, 2010). Other benefits of agroforestry include more favorable microclimates with enhanced biodiversity and reduced wind velocity, enhanced suppression of insect pests and weeds, decreased levels of soil erosion, increased water infiltration, improved production potential by increasing crop yield, and diversification of production by generating products from the intercropped trees.

Typically, agroforestry is associated with farming in tropical and subtropical arid regions. However, there are also opportunities for agroforestry in temperate regions (Smith *et al.*, 2013). The challenge lies in the political will to promote the research required to the adoption of agroforestry as a mainstream agricultural management approach. A number of plant biotechnology innovations are ready to boost agroforestry systems, including trees with a variety of GM traits (ISAAA, 2017).

### Future food system

Our food system is highly dependent of agriculture. Crop production and livestock provide the vast majority of the diverse, safe, and nutritious foods we need. But the challenge of delivering food and nutrition security for one and all in a sustainable way goes beyond agriculture. It requires wider transformation of the entire food system, from production to consumption. Starting by consumption behaviors: reducing the over-consumption of calorie-dense food will improve the overall sustainability of agriculture and food systems, whilst addressing a major threat to health. Globally, more than 1.9 billion adults, over 340 million children and adolescents aged 5-19, and 41 million children under the age of 5 were overweight or obese in 2016 (WHO). Obesity is often associated with low income. A healthy diet has become more expensive and, for poor people, food rich in sugar, fat, and salt is often more accessible than nutritious food. For these people, overconsumption of calories coexists with malnutrition in terms of micronutrients. Obesity is a risk factor for several diseases such as non-communicable diseases (NCDs), diabetes, heart disease, and cancer, with significant consequences for both individual health and public health.

Another issue that needs to be addressed is where the food is produced. Although there is a consensus that per capita agricultural production has outpaced population growth, food is not produced where it is mostly consumed or needed. In 2017, one in nine persons in the world suffered from some form of hunger. The number of stunted children is still “unacceptably high”, in view of the target to reduce stunting by 40% by 2025 (FAO, IFAD, UNICEF, WFP and WHO, 2018). Amelioration of crops that are essential parts of the diet of those who live in poor arid regions can have a major impact on food security. Crops indigenous to these regions have the potential to mitigate the impact of climate change on food production, because they tolerate fluctuations in growing conditions and are resistant to local diseases and pests. Compared to the world’s major crops, indigenous crops are better adapted to their niche environments. However, many of these local varieties are being abandoned by farmers in favor of major crops that are sometimes promoted even in less suitable areas (Chivenge *et al.*, 2015). Now, triggered by concerns about climate change and the sustainability of food production, these “neglected and underutilized species” or “orphan crops” are receiving the attention they deserve from the research community. Public sector and public-private partnerships, particularly in Africa, are advancing research on orphan crops for nutrition and resilience (Pretty *et al.*, 2014; Ricroch and Hénard-Damave, 2016; De Buck *et al.*, 2016).

Another major hurdle of the current food system is food wastage. According to FAO “*Roughly one third of the food in the world produced for human consumption every year —approximately 1.3 billion tons — gets lost or wasted*.” Global loss or waste estimates are: 30% of cereals production, 20% of dairy products, 35% of fish and seafood, 20% of meat, 20% of all oilseeds and pulses, and 45% of roots, tubers, fruits and vegetables (FAO, 2013). These values are important. Minimization of food losses could indeed be of great help in achieving global food security. A number of GM traits have been developed that improve food storage stability in crops, e.g., oxidative browning was reduced in potatoes and apples by downregulation of the polyphenol oxidase gene (Bachem *et al.*, 1994; Maxmen, 2017); GM tomato with high-level polyphenol accumulation presented extended shelf life and decreased susceptibility to grey mould, *Botrytis cinerea* (Zhang *et al.*, 2013); actually the first commercialized transgenic product, tomato FLAVR SAVR was designed to delay fruit softening by silencing of the polygalacturonase gene.

Food loss or waste arises at all phases of the food supply chains, from harvest, post-harvest, and processing, to marketing, retail, and consumption stages. The uneaten food accounts for about 8% of the greenhouse gas emissions. The pattern of food waste is different between high- and low-income regions. While in high income countries food waste is higher at the processing, distribution, and consumption stage, in low income countries, food losses occur primarily at production and post-harvest phases, i.e., from harvesting to marketing (FAO, 2013).

Developed regions, such as the European Union (EU), are more concerned in reducing food waste at an extended post-harvest stage, i.e., losses between harvest and the time of consumption. These are substantial losses, ranging from 5-10% to more than 50%, depending on output and geographical area. The causes of the loss vary from improper or inadequate handling, threshing, drying, cleaning, or processing, or because of faulty or deficient storage, transporting, or packaging of the food (Global Knowledge Initiative, 2017). Strategies to solve these problems are relatively simple and require no or few innovative inputs. They are considered “low hanging fruit” and are being prioritized in EU countries. This approach can also be useful for low income countries. Public and private sectors are cooperating to bring practices, protocols, and cold-chain equipment to less developed countries. However, we cannot forget that low-income regions suffer more from losses at stages of harvest and strict post-harvest, i.e., between the harvesting and marketing. Plant biotechnology innovations that could bring solutions to reduce losses during harvest and post-harvest are being overlooked because “*it is still a sensitive subject with no political clarity*” (Global Knowledge Initiative, 2017). This is a regrettable mistake.

## The role of science in a global and intercultural world

In my view there are three major forces that shape our modern world: science, economy, and society. These forces are intertwined and interdependent. Science is the driver of innovation, which in turn is the central force of a society’s economic transformation. Society defines science priorities and pushes innovation forward (or backward). Our modern multicultural society is fashioned by individuals whose attitudes are shaped by core values of what is considered good or bad, acceptable or unacceptable, desirable or undesirable. Values are learned ideas that are molded by different forces, including family, history, education system, religion, media, and economics. Core values of a culture do not change quickly or easily, they are passed on from generation to generation. Paraphrasing Haidt (2012) core values `bind and blind’. Beyond behaviors and practices that are apparent to the casual observer (e.g., language, food, flags, festivals, and aesthetics), core values shape the concept of self, morality, beliefs, and the decision-making capacity of an individual. I wonder if educational systems have been efficient in drawing attention to the value of scientific reasoning in shaping core values.

In debates about the proper place of science in society, we often hear arguments that the scientific method has its limits and that methods employed in the humanities or in philosophy are the best tools to understand society (Boudry and Pigliucci, 2017). I sustain the controversial view that scientific reasoning is the only worthwhile mode of inquiry and, by its very essence, it cannot overstep its proper limits. Scientific reasoning is built upon principles of re-evaluation and questioning of authority. Scientific progress hinges on continual discovery and the extension of previous discoveries. When a new and better methodology is established, a prevailing hypothesis is questioned again in the light of new tools. Scientific theories change with adequate reasoning and verifiable evidence, and previous discoveries serve as the basis for subsequent breakthroughs. So, if a given problem cannot be approached by verifiable evidence, science does not have a word to say. But it is never definitive. If a new method is suitable to revisit a given empirical hypothesis, science is up to tackle the subject. In that way, as science progresses and new techniques are developed, science, without overstepping its limits, can study disciplines that before were approached only through empirical knowledge.

Indeed, I argue that scientific methods used in the life science can bring great contribution to the social sciences, as the latter deal ultimately with human beings. The enormous progress that we are witnessing in neurobiology and cognitive sciences is unravelling the mystery of consciousness and is starting to reveal how nature and nurture shape our feelings and the making of cultures. This realization had already begun by the end of the nineteenth century. Several scientists, among them, Darwin, James, Freud, and Durkheim have acknowledged the role of biology in the shaping of cultural events. The field of evolutionary psychology is now shedding new light on the biological transmission of culture-related traits. (Damasio, 2018).

Compelling scientific evidence in neuroscience and psychology indicate that emotions play a major role in decision-making. Patients suffering injuries in the ventromedial prefrontal cortex, which is involved in the interaction of emotion and cognition, have both reduced abilities to feel emotions and difficulty in making optimal decisions (Damasio, 1994; Bechara *et al.*, 1999). The psychological field of affective science have provided strong evidence that emotions influence the processes of decision making in a manner that is neither random nor epiphenomenal, and that emotions constitute powerful and predictable drivers of decision making. Current opinion among psychologists suggests that emotions influence decision-making via changes in both the content and depth of thought, as well as in the content of implicit goals (reviewed by Lerner *et al.*, 2015). Emotions *per se* are probably not detrimental to decision-making; sometimes they are helpful. Whether a specific emotion ultimately improves or degrades a specific judgment or decision depends on how it interacts with the processes of thinking. One current theory proposes that human decision-making operates in two parallel but linked thinking processes – System 1 and System 2. System 1 operates very quickly with little conscious awareness. Generally, this method consists of systematic simplifications and deviations that are largely on the basis of pattern-matching, associative memory, assumptions, and emotion. Most of the time we use System 1, as it helps us to gather information quickly, being very useful for managing the sheer number of decisions we take daily. The System 2 cognitive process is slower, more logical and deliberative. It recalls previous information and weighs the strength of variables before coming to a decision (Kahneman, 2012). Emotions are elicited rapidly and can trigger swift action, consistent with System 1. But some emotions (e.g., sadness) can trigger System 2.

I like to call the mental shortcuts that help us gather information and make decisions quickly as our ‘innate intelligence’ by analogy to ‘innate immunity’. Innate intelligence is experienced but not well studied. Innate intelligence is based on ancient knowledge, which is not acquired through experience. Ancient knowledge is our innate ability of combining pattern-matching and associative memories with feelings generated by negative. as well as positive emotions. Innate intelligence was crucial to the survival of early humans because it assisted our species in finding food and recognizing predators in savannas and jungles. Today, our innate intelligence can be a handicap when we have to make decisions on how to keep our society nourished, healthy, and at peace whilst maintaining the Earth System within its boundaries.

The human thinking system is part of human biology and cannot be updated as easily as computers operating system, at least not with today’s scientific knowledge. We cannot have a healthy life without emotions. But we can try to keep our thinking processes out of the control of emotions, so that our decisions are less prone to biases. This is indeed critical in our `post-truth society’ where emotion and personal beliefs are more influential in shaping public opinion than are objective facts. Peoples’ fears are being manipulated by dishonest people who are using digital tools to spread superficial and fake news at a scale and speed never seen before. One dramatic example is the reckless spread of false claims and half-truths of today’s populists, which is damaging the democratic system in Europe and in the Americas (Wodak, 2015). People’s attitudes towards plant biotechnology is another example on how dishonest activists and problematic scientific dissidents are tapping into people’s emotion and intuitive preferences to spread unsubstantiated negative representations of GMOs (Blancke *et al.*, 2015). This is not only a challenge for scientists, but also for policy makers and society as a whole.

Irrational feelings are nourished by ignorance, which is an easy prey to intellectual dishonesty. When ideological views are contradicted by the consensus of scientific opinion regarding the evidence, it is all too common for ill-informed people to reject the science, particularly if they have been under the pressure of a massive marketing campaign (Van Montagu, 2016). Decisions on the use of technologies must be taken on the basis of serious scientific peer-reviewed analysis of risks and benefits. If society cannot make factual decisions, monetary greed, ideological dogmas, and myths will take over.

## Concluding remarks

It is acknowledged that the degraded state of nature is the outcome of the dramatic population increase. There we are, and there is no way back. Human ingenuity is the cause, human ingenuity will have to find the remedies. Science has provided many tools to help humanity to reduce global environmental risks and promote global sustainability. But science alone will not solve problems and shape the future. The three pillars that sustain human civilization – society, science and the economy - must be in correct symmetry and based upon solid ethical and moral grounds; there is no place for nationalistic, xenophobic, racist, and anti-science rhetoric.

We have witnessed, in the case of plant biotech in Europe, situations that illustrate the imbalance of the three pillars and the poor quality of the soil of values on which our societies rests: Society, through governments, financed knowledge creation in public research institutions, which allowed industry to deliver GM crops to the market. But while industry favored products that were the most profitable for the commercial producer, numerous innovations with clear benefits to the public in general where kept on the shelf at public institutions due to lack of industrial partners. In a number of cases, industry acquired start-ups, frequently spin-offs of public sector research, with the purpose of phasing out innovations that could compete with industry’s own products. The lack of products that bring clear benefit to the consumer generated uneasiness among the public, which was susceptible to campaigns, claiming that GM products were not safe, and asking for strict regulations for planting and marketing GM foods. This severely limited others from entering the market because of expensive or impossible hurdles to the entry of new GM products. Only the holders of the patents to the few early GM products were able to continue to sell their few products. The consequent monopoly of GM seeds by transnational agrobusiness companies accrued the campaign of ideology groups, which in turn lobbied with politics to ban the cultivation of GM crops in EU. The political decision triggered the shrinking of public funds for research on plant biotechnology, severely delaying the development of innovations that are badly needed to promote agricultural sustainability.

The above example shows that the same technology that fosters development can also become the source inertia. Avoiding this trap will demand a more enlightened society, able to make its choices on the basis of facts strongly supported by scientific reasoning, not fiction. Our world will continue to evolve. Human progress is inevitable. Today, the whole social arena, be it legal, economy, food system, healthcare, energy, education, etc., is wide open to revolutionary developments. The so-called disruptive technologies, in particular infotech and biotech, have a huge transformative potential. But advances must be accompanied by adaptations in the social, political, and cultural arenas in order to attain the paradigm shift our society calls for. There is a need to reconsider the relationship between market, state, and society. As observed by Mazzucato (2018), it is critical to acknowledge that it is society through government, not private business, that finances fundamental research and education. The state has often been a tremendous force for technological innovation and radical risk taking. With the financial sector outpacing the growth of industry, and industry maximizing shareholder values at the expense of society, we misidentify who really creates value. Scientific knowledge must be deployed to help in the construction of a better and inclusive future, with healthy and stable economies, fair and well-governed societies, respect for human rights, respect for the environment, and consequently world peace.
